# Spectral Features of Heart Rate Variability in Williams Syndrome During Sleep

**DOI:** 10.3390/jcm15114317

**Published:** 2026-06-03

**Authors:** Bence Schneider, Ferenc Gombos, Ilona Kovács, Róbert Bódizs

**Affiliations:** 1 Institute of Behavioural Sciences, Semmelweis University, 1085 Budapest, Hungary; bodizs.robert@semmelweis.hu; 2 HUN-REN-ELTE-PPKE Adolescent Development Research Group, 1088 Budapest, Hungary; 3 Laboratory for Psychological Research, Pázmány Péter Catholic University, 1088 Budapest, Hungary; 4 Institute of Psychology, ELTE Eötvös Loránd University, 1064 Budapest, Hungary; 5 Institute of Cognitive Neuroscience and Psychology, HUN-REN Research Centre of Natural Sciences, 1117 Budapest, Hungary

**Keywords:** heart rate variability, Williams syndrome, spectral analysis, fractals

## Abstract

**Background:** This study analyzed spectral alterations of heart rate variability (HRV) in Williams syndrome (WS) during sleep, taking into account the multi-fractal properties of RR-interval spectra, including effects of aging and sleep structure. **Methods:** Using ECG recordings of 20 subjects with WS and matched typically developing (TD) controls, fractal and oscillatory spectral components of RR-intervals were computed. The fractal component was parametrized with a piecewise-linear function, allowing a breakpoint and separate slope and intercept values in the lower- and higher-frequency domains. The dominant peak frequency and prominence were extracted from the LF (0.04–0.15 Hz) and HF (0.15–0.4 Hz) bands. **Results:** Strong WS/TD group differences were found in the breakpoint frequency, high domain slope, intercept and HF peak prominence. The LF peak frequency showed a slight age-dependent decrease only in TD, and reduced values in WS independent of age. Principal component analysis identified a main fractal component describing typical alterations in the spectrum in WS, which exhibited sleep-structure associations. **Conclusions:** The broken power-law model successfully characterized the fractal component of RR-interval spectra, capturing altered cardiac regulation in WS, while suggesting the fractal parameters as possible biomarkers of the degree of general autonomic deregulation.

## 1. Introduction

Williams syndrome (WS, also known as Williams–Beuren syndrome) is a multisystem congenital genetic disorder caused by the microdeletion of the chromosome region 7q11.23. It is characterized by mild intellectual disability, developmental delay, characteristic facies, and altered cognitive abilities, including enhanced short-term memory and verbal skills, but deficits in visuospatial cognition. Individuals with WS exhibit some specific personality traits like excessive empathy and social disinhibition and are often comorbid with ADHD and generalized anxiety disorder [1]. Atypical development in WS can be manifested as early onset of puberty, premature graying of the hair, high frequency hearing loss, cataracts, etc., all indicating accelerated aging. More than half of WS patients are affected by sleep disorders, specifically by difficulties in sleep initiation and maintenance, decreased total sleep time and sleep efficiency, while intra-sleep wakefulness and daytime sleepiness are more frequent. The acceleration of age-related sleep deterioration was also observed in several sleep quality indices in WS compared to typically developing subjects [2].

The prevalence of cardiovascular diseases is high (80%) in WS, being the largest cause of mortality, with aortic stenosis forming the most typical condition due to the reduced elastic properties of vessel walls as a consequence of elastin insufficiency inherent to the syndrome [3,4].

Even in the absence of severe pathologies, alterations in cardiac regulations can be present, e.g., different arrhythmias were identified without being associated with structural cardiac disease [5]. The coexistence of dysregulations in sleep [2,6,7], cardiac activity [3] and affective functioning [8] suggests a general involvement of the autonomic nervous system (ANS) in the symptoms of the syndrome. Atypical ANS functions are evidenced by altered heart rate reactivity and electrodermal activity in response to affective social stimuli [8], no arousal level synchronization with others, indicated by a lack of pupil size contagion [9], as well as diminished circadian modulation in the heart rate and a general reduction in heart rate variability (HRV) indices, observed in 24 h ECG recordings of WS subjects [10]. Moreover, recent evidence suggests a fragmentation of heart rates in WS subjects on a short time scale, involving erratic behavior characterized by relatively frequent beat-to-beat accelerations and the related emergence of inflection points. Authors suggest the potential relevance of non-autonomic cardiovascular modulators in the emergence of this picture [11].

The HRV index had been considered to reflect changes in the ANS, with the low frequency (LF) 0.04–0.15Hz component corresponding to sympathetic and the high frequency (HF) 0.15–0.4Hz component to vagal activation traditionally. Newer studies suggest that LF power is affected by both sympathetic and vagal activation [12]. Besides cardiovascular diseases, changes in HRV have been identified in conditions known to be associated with WS, among which reduced HRV-indicated autonomic dysregulation in ADHD and anxiety disorders [13,14] are of potential interest. Whereas a recent meta-analysis revealed alterations in HRV of subjects living with various neurodevelopmental conditions, including autism spectrum disorder and ADHD [15], the evidence regarding the alterations in cardiac autonomic indices of ANS or cardiological dysfunctions in WS is scarce. In the context of accelerated aging in WS, the HRV parameters might also provide relevant information, as it was proposed that HRV is not only associated with chronological age, but potentially an organismal-level biomarker of processes that contribute to aging, and thus it is able to reveal trajectories of unhealthy aging [16] or perhaps even the premature death of WS subjects [10]. Despite the large overlap between the specific sensitivity of HRV indices and WS physiopathology, aspects of the syndrome are under-explored in the HRV analysis framework. The gap in the field is specifically evident in terms of a lack of reports on sleep-related HRV alterations of WS subjects. Sleep-related HRV analyses indicate varying levels and types of ANS dysregulation reported to be independently associated with both disordered sleep and mood- and anxiety-related disorders [17], whereas ADHD without sleep disorders did not affect cardiac vagal activity in this dormant state [18]. While heart rate is strongly influenced by physical and psychological activity, food intake, and other environmental factors during wakefulness, sleep opens a window on purely endogenous autonomic regulation, devoid of external stimuli, in lack of major postural changes, amplifying the clinical relevance of HRV measurements. As PSG recordings commonly include ECG derivations from which HRV measures can be easily calculated, these recordings represent clinically valuable information for automated screening [19].

Apart from autonomic control, the heart rhythm is modulated by many factors ranging from the molecular scale to the organ level [20]; as such, the RR-interval time-series comprises a complex, fractal component, which is manifested as a power-law in the power spectral density (PSD) of the time-series [21]. The exponent of the power-law, or the so-called spectral slope, characterizes the persistence and long-term memory of the signal; it was found to carry meaningful information about electrophysiological signals, such as local field potentials, electrocorticograms, electroencephalograms, and magnetoencephalograms [22,23]. The aim of our study is to identify spectral alterations in HRV due to Williams syndrome in sleep, using a parametric model that takes into account the underlying multifractal nature of RR-interval time series, which has been identified as a property of healthy heart rate dynamics [24,25]. Furthermore, to explore the association between sleep structure and the extracted spectral parameters.

## 2. Materials and Methods

Bipolar ECG derivations were analyzed from an ambulatory polysomnography database [6] containing whole-night home sleep recordings (recording lengths range: 6.66–12.19 h, mean: 10.36 h) of 20 subjects with WS (13 females, age range: 6–29 years, mean: 19.6 years) and an age and sex matched control group of 20 subjects (14 females, age range: 6–29, mean: 20.3 years). All participants had given their written consent to participate in the data recording; the procedure had been approved by the Ethical Committee of the Budapest University of Technology and Economics, in line with the Declaration of Helsinki. Recordings were performed by using a 32 channel SD-LTM Hardware together with the BRAIN QUICK SystemPLUS EVOLUTION v1.02 software (Micromed, Rome, Italy). Signals were high-pass filtered at 0.33 Hz and low-pass filtered at 1500 Hz by a 40 dB/decade anti-aliasing hardware input filter. Data were collected with 22-bit resolution and with an analog-to-digital conversion rate of 4096 Hz/channel (synchronous). A further 40 dB/decade anti-aliasing digital filter was applied by digital signal processing, which low-pass filtered the data at 124 Hz. After this, the digitized and filtered EEG was subsequently undersampled at 1024 Hz. A 50 Hz digital notch filtering performed by the recording software was also used. R-peak detection was carried out automatically on the bipolar ECG channel, and then the RR-interval time series was extracted for each subject using the Kubios HRV software, version 4.1.2.1 (Kubios Oy, Kuopio, Finland) [26]. Next, the Irregular-Resampling Auto-Spectral Analysis (IRASA) method [27] was applied to the entire time series in the frequency range of 0.001–0.5 Hz, in order to separate the fractal and oscillatory PSD components, using a moving window of 4096 s.

Sleep structure indicators were available from the original study that included total sleep time, sleep efficiency, wake after sleep onset, sleep latency, and relative durations of each sleep stage (W, N1, N2, N3, R).

### 2.1. Spectral Parametrization

**Figure 1:**
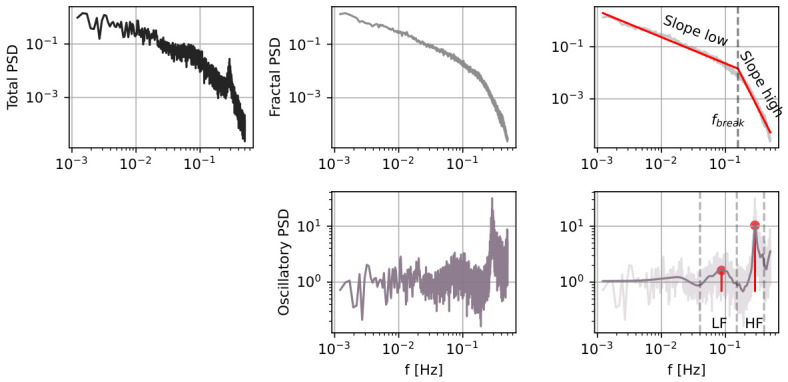
Outline of the spectral parametrization. The original PSD (**top left**) is split into the fractal and oscillatory components (**middle column**), then the fractal component is fitted with a piecewise linear function (red line), allowing for a custom breakpoint (grey dashed line) that separates the linear fits in the low- and high-frequency domains (**top right**). Peak detection is applied in the LF and HF bands to the oscillatory component after Gaussian smoothing, and the frequency and prominence of the dominant peak (red indicators) are extracted from both bands (**bottom right**).

As the fractal component exhibits a broken power-law relationship between the spectral power density and frequency, a piecewise linear function was fitted in the log-log domain, allowing for a custom breakpoint, and two slope and intercept values in the lower and higher frequency domains. The oscillatory component was defined as the log-log domain difference in the total PSD and the fractal component with Gaussian smoothing applied (the standard deviation of the kernel was 20 samples). The peak frequency and prominence of the dominant peak were extracted by identifying local maxima from the LF (0.04–0.15 Hz) and HF (0.15–0.4 Hz) bands, using the “find_peaks” function from the scipy.signal python library, Scipy v1.10.1, Python 3.8.3, see Figure 1.

The spectral approach is especially suitable for analyzing whole-night HRV recordings, as it captures information about several time-scales in a single function, which can then be further reduced into a small set of non-redundant parameters. We consider our methodology to be an extension of traditional band-based spectral HRV techniques, as it operates in the frequency domain and can be focused on canonical frequency bands (LF, HF in our case); however, power-spectral values are not simply summed into a band-power value but are separated into contributions of fractal and oscillatory activity, which could distinguish different underlying physiological phenomena.

### 2.2. Statistics

A general linear model was assessed for each spectral parameter as the dependent variable, including possible main effects of group (TD/WS), age and group*age interaction. Correlations between the spectral parameters and age were also calculated for each group separately. As we revealed significant correlations between the parameters describing the fractal component, principal component analysis (PCA) was carried out to reduce the dimensionality of the data. Furthermore, the number of analyzed sleep structure indicators was relatively large (nine in total), some of which were (by definition) correlated; thus, PCA was applied in their case as well. As the dimensionality reduction proved to be meaningful both for the spectral and sleep structure parameters, associations between the two sets of principal components were further examined.

## 3. Results

The broken power-law fitting was successful, and the mean goodness of fit was <$R^{2}$> = 0.990 (range = 0.962–0.997, SD = 0.007). Peaks were detected in all control subjects in both bands, whereas in the WS group, 18 and 17 peaks were found in the LF and the HF bands, respectively (out of the total 20 spectra; see Table 1 and Table 2 for more descriptive statistics).

### 3.1. Effects of WS Group, Age and Interactions

Strong group effects were found in some of the fractal parameters, namely, the high domain slopes were flatter ($F_{G}=30.07,\;p<0.001$), high domain intercepts increased ($F_{G}=20.07,\;p<0.001$), and the breakpoint frequencies were decreased significantly ($F_{G}=21.02,\;p<0.001$) in WS subjects compared to controls. An attenuation of HF peak prominences in WS ($F_{G}=13.23,\;p<0.001$) was also observed.

**Figure 2:**
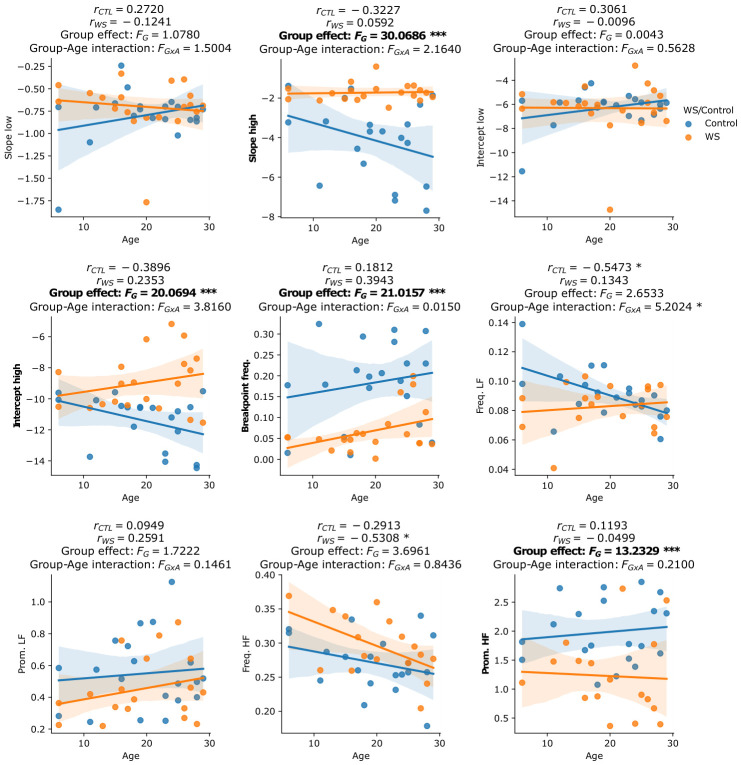
Effects of WS/TD group and age on the spectral parameters. Within-group Pearson correlations with age are displayed above each plot for controls ($r_{CTL}$) and WS ($r_{WS}$) separately, as well as a general linear model result with main group effect statistic ($F_{G}$) and the statistical power of the group*age interaction ($F_{GxA}$). Significance marked as: * p<0.05, *** p<0.001, boldface—significant after Benjamini–Hochberg correction with Q = 0.05 (N = 40 subjects, 20 WS).

A moderate slowing of LF peak frequency with aging (r=−0.55, p<0.05) was suggested in the control population, which was not present in the WS group (r=0.13, n.s.), however. When assessing the correlations within the group, this difference was also suggested by the group*age interaction in the general linear model ($F_{G*A}=5.20,\;p<0.05$); however, the significance of these age effects did not withstand corrections for multiple comparisons; see all effect sizes of all spectral parameters in Figure 2.

### 3.2. Fractal Parameters PCA

**Figure 3:**
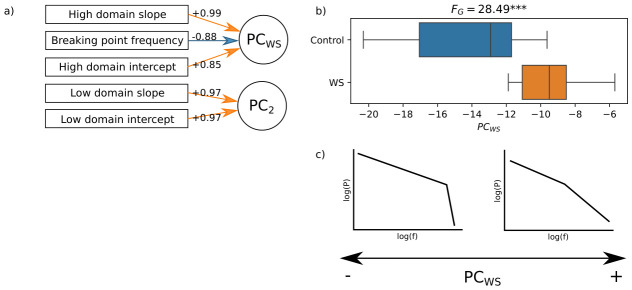
Principal component analysis in the fractal parameters. Due to the high correlation of the original parameters, the main WS vs. TD group differences in the fractal spectra can be described by a single component $PC_{WS}$: (**a**) contributions of the fractal parameters to the two principal components, (**b**) the first component shows a significant effect of WS (*** p<0.001), (**c**) characteristic changes in the power spectrum explained by the $PC_{WS}$ component, WS exhibiting increased high domain slope and intercept, furthermore decreased breakpoint frequency. The orange arrows mean positive, blue negative contributions.

A high degree of correlation was found between specific pairs of fractal parameters (Table 3), which indicates that the variance in the data could be described by fewer parameters. In order to reduce the dimensionality of the fractal parameter space, PCA was applied, which resulted in two principal components. The first principal component was a linear combination of the high domain slope, high domain intercept and breakpoint frequency, while the low domain slope and intercept contributed to the second principal component. Assessing the principal components as dependent variables in a general linear model as before, the main effect of WS/TD group was identified in the first component ($F_{G}=28.49,\;p<0.001$), suggesting that WS-specific alterations in the RR-interval spectra are characterized by a joint increase in the high domain slope and intercept, along with a decrease in the breakpoint frequency, and as such, we will refer to this principal component as a WS-related component, $PC_{WS}$; see Figure 3. The second component was a linear combination of the low domain slope and intercept, which did not show effects of WS or age.

### 3.3. Sleep Structure PCA

**Figure 4:**
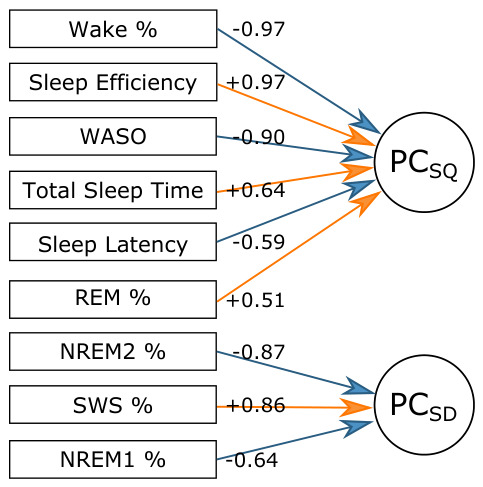
Principal component analysis of sleep structure indicators, in which two main components were identified. Higher values of the first component could be associated with better sleep quality $PC_{SQ}$, while the second increased with deep to light sleep duration ratio $PC_{SD}$. The orange arrows mean positive, blue negative contributions.

**Figure 5:**
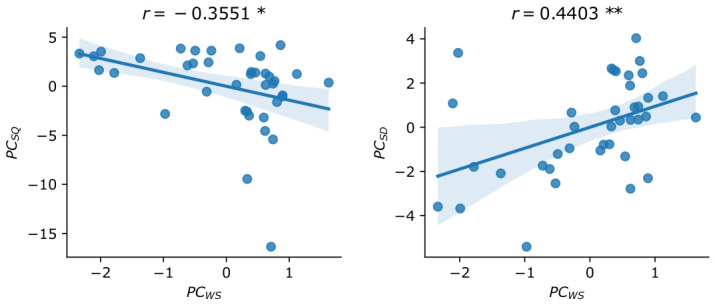
The $PC_{WS}$ component is correlated with the principal components of the sleep indicators; increased $PC_{WS}$ scores imply worse sleep quality and a shift toward deeper sleep. * *p* < 0.05, ** *p* < 0.01, N = 40 subjects.

Many pairs of the nine sleep structure indicators were also significantly correlated (15 out of 36 total pairs), which were reduced by PCA to two components ($\chi^{2}=1007.89$, p<0.001). The first component had positive contributions of sleep efficiency, sleep duration and relative REM duration and negative contributions of relative wake duration, wake after sleep onset and sleep latency, so it can be termed as a general sleep-quality component $PC_{SQ}$. The second component was positively associated with relative SWS duration and negatively with relative NREM2 and NREM1 durations, and thus an indicator of deep versus light sleep duration ratio, referred to as sleep depth component $PC_{SD}$; see Figure 4. After the reduction in the parameters, the correlations of the $PC_{WS}$ component were assessed with the $PC_{SQ}$ and $PC_{SD}$ components. Higher Williams component scores were associated with significantly lower sleep-quality $PC_{SQ}$ components (r=−0.355, p<0.05) and increased $PC_{SD}$ sleep depth component values (r=0.440, p<0.01); see Figure 5.

## 4. Discussion

One of our main findings was that the parameters of the broken power-law model describing the fractal component of the HRV spectrum were altered in WS. More specifically, the breakpoint frequency was reduced (shifted toward the lower frequencies), and an increase in the slope (flattening of the high-frequency spectra) and intercept above the breakpoint were revealed. An apparent contradiction between our findings and the general power reduction reported in a previous study [10] might arise; however, we note that there were no significant group differences in the fractal parameters below the breakpoint, and slopes above it were steeper in general in both groups (see Table 1). Thus, lower breakpoint frequencies imply less total power. At the same time, the mean breaking frequency in WS was at 0.06 Hz, well below the upper boundary of the LF band (0.15 Hz), meaning that the changes in the fractal component can explain power reduction both in the LF and HF bands. In addition, the attenuation of HF peak prominence can also be responsible for the increased LF/HF ratio reported in the same study.

We hypothesize that the phenomenon of accelerated aging [2,6] could be reflected in the LF peak frequency, as it showed deceleration with age in the control group, but generally lower values regardless of age in WS, also indicated by the interaction term in the general linear model; however, these results were limited by small sample size and high signal-to-noise ratio. The findings could suggest a subtle alteration in the Mayer wave sinus arrhythmia [28] of WS subjects during sleep, but revealing the physiological bases of this subtle alteration characterized by age-independent and lower frequency oscillations in the “human Mayer range” needs further studies.

We propose that the flattening of the high-frequency spectral slope could reflect the erratic, beat-to-beat type fragmentation of heart rate in WS subjects reported recently [11]. Indirect evidence supporting this assumption can be based on the fact that heart rate fragmentation is quantified on the basis of the relative density of inflections in the RR-series. Frequent inflections indicate frequent short-scale changes in the time series, which are known to be reflected in a flatter spectral slope. More specifically, the nominal value of the group mean of high-frequency spectral slope in WS subjects (−1.722) falls in the so-called antipersistent range (>−2), whereas in the TD participants, the same value (−4.168) is definitely below the threshold of −2, indicating a persistent time series. The former reflects rapid shifts in the heart rate increments (decreases tend to be followed by increases and vice versa), whereas the persistent spectra imply a positive correlation in the TD subjects (increases tend to be followed by further increases and vice versa) [29]. Present results suggest that bifractal spectral examination of heart rate is potentially indicative of the erratic cardiovascular dynamics in WS, which were formerly proposed to reflect the effects of non-autonomic cardiovascular conditions [11].

In addition to the alterations of fractal HRV spectra outlined above, the decrease in HF peak prominence in WS subjects was also revealed. Decreased HF peak prominence indicates a reduction in respiratory sinus arrhythmia and, thus, a possibly related reduction in vagal modulation of the heart rate of WS subjects during sleep. Together, these findings indicate that the heart rate regulation during human sleep is a result of a complex combination of bifractal and oscillatory processes. Former studies revealed that mental and cardiovascular stress, including baroreceptor stimulation, advancing age, as well as compromised cardiac function, reduce respiratory sinus arrhythmia [28]. As a consequence, the sleep-related reduction in the spectral peak of HF HRV in WS is indicative of the above-mentioned conditions, among which advanced age and compromised cardiac functions are of special interest from the perspective of our present focus. Given the fact that our typically developing participants were age-matched with the WS subjects, the dampened spectral peak-indicated reduction in respiratory sinus arrhythmia during sleep supports the advanced aging concept of WS. Moreover, cardiac comorbidities are particularly frequent in WS subjects. The combination of the erratic, beat-to-beat fluctuation in heart rate indicated by the flattened high-frequency spectral slope with the decreased respiratory sinus arrhythmia is an exemplary case of the intertwined nature of cardiac and vagal factors in shaping the HF spectral profile of HRV [28], which was formerly not reported in WS. The combined alteration of the respective measures in WS suggests that autonomic and cardiovascular modulators interact in shaping the heart rate dynamics of WS subjects during sleep.

Moreover, the present results further strengthen the pluripotent clinical value of polysomnographic studies. The latter involve an ECG derivation, and thus, are ideally suited for revealing the short- and long-term dynamics of resting heart rate during sleep in various clinical conditions and research settings [19].

As the principal component analysis successfully reduced the correlated fractal parameters to only two components, a general feature could be identified in the HRV spectra, typical of individuals with WS. Furthermore, this WS component was negatively correlated with sleep quality indicators and positively associated with higher deep to light sleep ratio, so we hypothesize that it is not only a feature of cardiac rhythm alteration, but of general deregulation in the autonomic nervous system, which couples sleep homeostasis with cardiovascular control, thus a potential biomarker for WS severity worthy of further investigation. As WS is a rare condition, the biggest limitation of this study is the small sample size, which covers a large age range, and while our results are statistically robust, and reproduce some expected effects, it remains a descriptive analysis, based on which we can hypothesize possible underlying mechanisms of autonomic deregulation in the syndrome.

## Figures and Tables

**Table 1:** Group-wisedescriptives of the broken power-law model parameters describing the fractal component. Parameters with significant group differences marked with boldface.

	Slope Low		Slope High		Intercept Low		Intercept High		Freq. Breakpoint	
	Control	WS	Control	WS	Control	WS	Control	WS	Control	WS
Valid	19	20	19	20	19	20	19	20	19	20
Missing	0	0	0	0	0	0	0	0	0	0
Mean	−0.804	−0.700	**−4.168**	**−1.722**	−6.298	−6.309	**−11.473**	**−8.968**	**0.183**	**0.068**
Std.	0.312	0.296	1.999	0.443	1.521	2.309	1.697	1.839	0.102	0.054

Freq. breakpoint—frequency of the spectral breakpoint, WS—Williams syndrome group.

**Table 2:** Group-wise descriptives of the extracted peak parameters from the oscillatory component in the LF and HF bands.

	Freq. LF		Prom. LF		Freq. HF		Prom. HF	
	Control	WS	Control	WS	Control	WS	Control	WS
Valid	19	18	19	18	19	17	19	17
Missing	0	2	0	2	0	3	0	3
Mean	0.090	0.083	0.548	0.453	0.269	0.295	**1.994**	**1.224**
Std.	0.018	0.016	0.240	0.203	0.041	0.044	0.559	0.696

Freq. LF/HF—frequency of most prominent peak in the LF/HF band, Prom. LF/HF- prominence of the dominant peak in the LF/HF band, WS—Williams Syndrome group, **boldface**—significant difference between group means.

**Table 3:** Pearson’s correlation matrix of all fractal parameter pairs with observations from all 40 subjects.

Variable	Slope Low	Slope High	Inter. Low	Inter. High
Slope high	0.059	—	—	—
Inter. low	0.914 *	−0.114	—	—
Inter. high	0.104	0.825 *	0.043	—
Freq. break	−0.011	−0.883 *	0.200	−0.538 *

* p<0.001, Inter.—intercept, Freq. break—breakpoint frequency.

## Data Availability

The raw data supporting the conclusions of this article will be made available by the authors on request.
